# Management of Refeeding Syndrome in Medical Inpatients

**DOI:** 10.3390/jcm8122202

**Published:** 2019-12-13

**Authors:** Emilie Reber, Natalie Friedli, Maria F. Vasiloglou, Philipp Schuetz, Zeno Stanga

**Affiliations:** 1 Department of Diabetes, Endocrinology, Nutritional Medicine and Metabolism, Inselspital, Bern University Hospital, and University of Bern, 3010 Bern, Switzerland; zeno.stanga@insel.ch; 2 Medical University Department, Division of General Internal and Emergency Medicine, Kantonsspital Aarau, 5001 Aarau, Switzerland; natalie.friedli@gmx.ch (N.F.); schuetzph@gmail.com (P.S.); 3 AI in Health and Nutrition Laboratory, ARTORG Center for Biomedical Engineering Research, University of Bern, 3008 Bern, Switzerland; maria.vasiloglou@artorg.unibe.ch; 4 Medical Faculty of the University of Basel, 4056 Basel, Switzerland

**Keywords:** refeeding syndrome, diagnosis, management, malnutrition, hypophosphatemia, nutritional support, nutritional therapy

## Abstract

Refeeding syndrome (RFS) is the metabolic response to the switch from starvation to a fed state in the initial phase of nutritional therapy in patients who are severely malnourished or metabolically stressed due to severe illness. It is characterized by increased serum glucose, electrolyte disturbances (particularly hypophosphatemia, hypokalemia, and hypomagnesemia), vitamin depletion (especially vitamin B1 thiamine), fluid imbalance, and salt retention, with resulting impaired organ function and cardiac arrhythmias. The awareness of the medical and nursing staff is often too low in clinical practice, leading to under-diagnosis of this complication, which often has an unspecific clinical presentation. This review provides important insights into the RFS, practical recommendations for the management of RFS in the medical inpatient population (excluding eating disorders) based on consensus opinion and on current evidence from clinical studies, including risk stratification, prevention, diagnosis, and management and monitoring of nutritional and fluid therapy.

## 1. Introduction

During World War II, many people suffered from hunger and starvation. Under these circumstances, Ancel Keys investigated the physical and mental effects of prolonged dietary restriction and the subsequent refeeding of 36 conscientious objectors in the Minnesota Starvation Experiment [1]. Most of the subjects experienced periods of severe emotional distress, depression, social withdrawal, isolation, decline in concentration, and decreases in metabolic rate, respiration, and heart rate. Several of the participants developed edema in their extremities. Later, at the end of World War II, further observations were made by Schnitker and Burger [2,3]. Numerous starving detainees developed severe symptoms such as heart failure, peripheral edema, and neurological disorders after a normal diet was reintroduced, and one of five died within the next few days [2,3]. Those observations led to the first description of the refeeding syndrome (RFS), almost 75 years ago.

To date, there is still no commonly accepted definition of RFS, and its detailed pathophysiology remains largely unclear. This is primarily due to the fact that the clinical manifestations of RFS are nonspecific, leading to RFS frequently being overlooked, underdiagnosed, and subsequently untreated. In the study of Hernandez-Aranda et al., up to 48% of malnourished inpatients developed RFS [4]. A sub-analysis of the just-published study of Schuetz et al. demonstrates that medical patients with confirmed RFS have significant mortality rates and increased non-elective hospital readmission, thus confirming the negative effect of RFS on clinical outcome [5,6].

Nutritional treatment is a central aspect of modern multimodal inpatient therapy. It aims to reduce complications and mortality rates, and to improve patients’ quality of life and autonomy [5,7]. Even though well tolerated, nutritional treatment has a potential risk of complications, including RFS, which is an exacerbated response to the metabolic change from a starvation to a fed state as a consequence of large amount of food in the replenishment phase. RFS is characterized by an imbalance of electrolytes (mainly phosphate, potassium, and magnesium), vitamin disturbances (e.g., vitamin B1 thiamine deficiency), and fluid imbalances, as well as limited organ functions, in some cases leading to mortality [8,9,10,11,12]. This article highlights, discusses, and reviews RFS in medical inpatients (excluding patients with eating disorders) in terms of pathophysiological aspects, preventive measures, clinical manifestations, risk evaluation, diagnostic procedures, and treatment methods.

## 2. Pathophysiology and Clinical Manifestations

**Figure 1:**
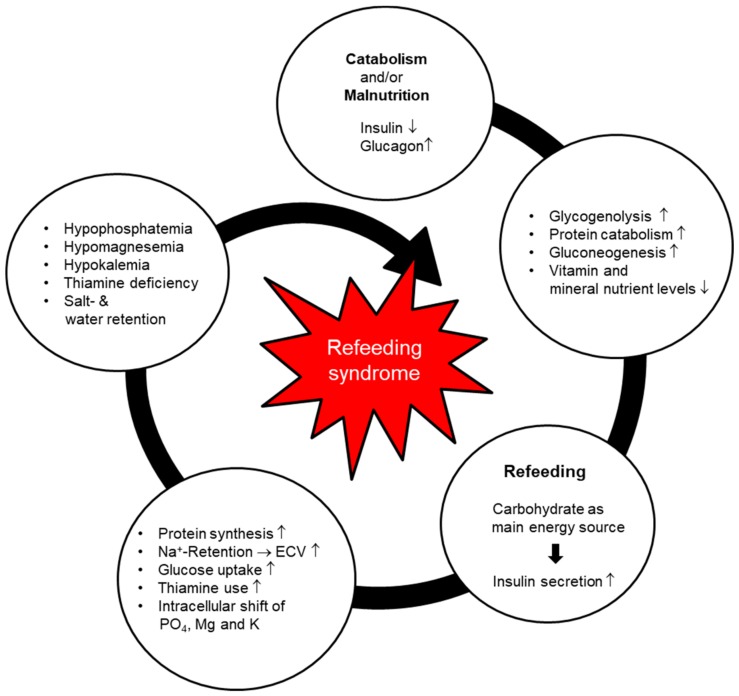
Pathophysiology of refeeding syndrome [22]. Used by permission of the Division of Diabetes, Endocrinology, Nutritional Medicine and Metabolism, Prof. Dr. med. Zeno Stanga (2019).

RFS is an exaggerated physiological response to glucose reintroduction (refeeding) after a prolonged phase of starvation or scarce food intake [13]. The precise pathophysiological mechanisms remain unclear, but recent assumptions are based on the processes described below (Figure 1).

In a catabolic state (due to reduced food intake or even starvation), insulin production is decreased, whereas glucagon and catecholamine are slightly stimulated [14]. During a fasting period, glucose oxidation is reduced and only takes place in the glucose-dependent tissues, such as the brain, renal medulla and red blood cells. The glycogen stores are reduced, leading to activation of gluconeogenesis and the production of glucose from endogenous amino acids, which are released by increased proteolysis. This process causes a reduction in muscle mass, thus inducing functional weakness and weight loss. Vitamin and electrolyte levels are decreased and stores are depleted [15]. After a few days, lipolysis increases, subsequently leading to raised levels of free fatty acids in the circulation. These free fatty acids stimulate ketogenesis in the liver, leading to high production of ketone bodies (in particular acetoacetate and beta-hydroxybuturate), which become the main suppliers of energy for the body [16]. During the catabolic state, metabolic processes are reduced to 30–50% of normal (adaptation phase) [13].

If balanced nutritional support with carbohydrates (refeeding) is introduced, glucose becomes the main energy supplier again, causing hyperglycemia and consequently an increase in insulin secretion. Anabolic processes are stimulated, leading to intracellular shifts of glucose, water, and electrolytes, and resulting in a potentially severe drop in serum micronutrient levels. The resulting electrolyte imbalances can cause life-threatening complications such as arrhythmia, spasms, or tetany [8,11,15,17,18]. Acid-base balance can cause significant electrolyte shifts and this needs to be considered as a differential diagnosis/contributing cause when suspecting refeeding syndrome (e.g., respiratory acidosis). A significant drop in phosphate, potassium, or magnesium levels may occur when the patient has been acidotic, and this is starting to resolve. As the intracellular shift of glucose is thiamine dependent, a deficiency in thiamine, as observed during catabolism, can lead to symptoms of beriberi. The more compromised the nutritional state, the higher the risk of RFS and the greater the severity of its manifestations [8,12]. There are many non-specific symptoms that potentially occur during RFS; the most commonly observed clinical symptoms in daily practice are tachycardia, tachypnea, and peripheral edema [8,15,19,20].

Clinical consequences due to electrolyte changes following increases in insulin include:

– Phosphate is an important electrolyte in the metabolism of macronutrients for both the energy production and transport processes. Phosphate is especially important in the refeeding phase, since glycolysis requires only phosphorylated glucose. Hypophosphatemia may cause several clinical manifestations, such as rhabdomyolysis, hemolysis, respiratory failure, and musculoskeletal disorders. Severe hypophosphatemia (<0.32 mmol/L) is considered a typical hallmark of RFS and in several studies is a central defining criterion [15,18].
– Potassium and magnesium are also important intercellular cations. Severe hypokalemia (<2.5 mmol/L) and/or hypomagnesemia (<0.50 mmol/L) may trigger potentially lethal arrhythmia, neuromuscular dysfunctions such as paresis, rhabdomyolysis, confusion, and respiratory insufficiency [15].
– Thiamine is an essential coenzyme in the metabolism of carbohydrates, allowing the conversion from glucose to adenosine triphosphate (ATP) via the Krebs cycle. When thiamine is lacking (human body stores last for approximately 14 days), glucose is converted to lactate, leading to metabolic acidosis. Thiamine deficiency may also lead to neurologic (Wernicke’s encephalopathy: dry beriberi) or cardiovascular disorders (wet beriberi) [15,16].
– Sodium: The major influence on the serum sodium level during the refeeding phase is the shift of sodium out of the cell as the potassium is pumped back into the cell (sodium-potassium-ATPase pump). In addition, the increased insulin level in the early phase of refeeding leads to sodium retention in the kidneys. Sodium concentration subsequently increases, thus inducing water retention. Noradrenaline and angiotensin II are stimulated and lead to augmented peripheral resistance and vasoconstriction [21]. This may cause peripheral edema and heart failure.

## 3. Current Level of Evidence

The current state of evidence for RFS was recently summarized in a systematic review by Friedli et al. [20]. It is mainly based on case series and retrospective, cohort, and case-control studies. To date, very few randomized controlled trials have been published. A recent experts’ consensus defined risk factors, occurrence, incidence rate, preventive measures, and treatment recommendations in medical inpatients [19]. A literature search for newly published studies was performed according to the criteria of Friedli et al. for the systematic literature review, excluding anorexia nervosa [20]. Due to the scarce evidence, the National Institute for Health and Care Excellence (NICE) guidelines on nutritional support in adults, containing recommendations on identification and treatment of malnutrition as well as management of nutritional therapy, are often used as the standard of care [23]. Consistent data on the management of RFS and adverse clinical outcome are largely lacking, and justify further research on specific preventive, screening, and treatment measures in patients at risk.

A secondary analysis of a large randomized controlled trial (EFFORT trial [5]) showing the beneficial effects of nutritional support in hospitalized patients provides evidence that, due to the consequences of RFS (higher mortality and non-elective readmission rates), patients at risk may benefit from a specific treatment [5,6]. This secondary analysis relying on the risk stratification and definition from the above-mentioned experts’ consensus [19] largely confirms the proposed risk factors and occurrence of RFS [6,8,12,19].

## 4. Prevention

### 4.1. Nutritional Support Teams

RFS is most likely to occur within the first 72 h after the start of nutritional therapy (replenishment phase), and to progress rapidly [20]. Quick recognition is crucial and requires well-trained medical staff [24]. In most hospitals, multidisciplinary nutritional support teams are available and assist the attending medical staff in the management of malnutrition. Such teams—consisting of physicians, dieticians, nurses, and pharmacists—contribute to improved quality and safety and optimal clinical outcomes [25,26,27].

### 4.2. Individual Risk Assessment

**Figure 2:**
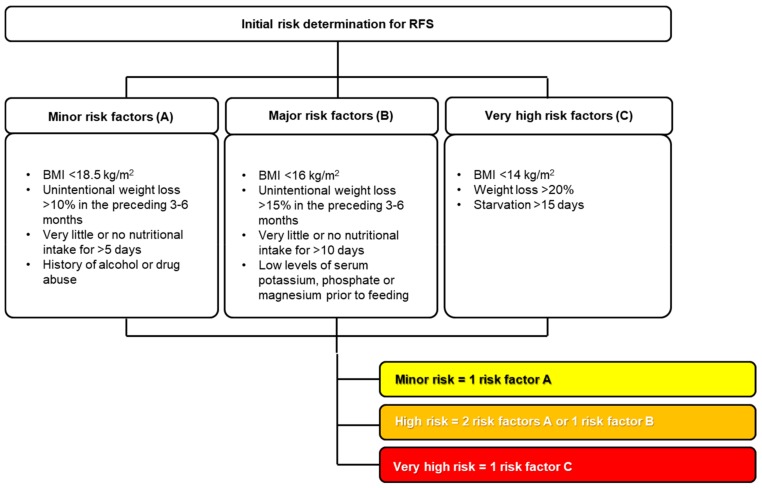
Risk stratification for RFS, according to [19,23]. This stratification has not been validated in a clinical trial [22]. Used by permission of the Division of Diabetes, Endocrinology, Nutritional Medicine and Metabolism, Prof. Dr. med. Zeno Stanga (2019).

Although RFS is associated with severe and potentially lethal complications, it is a preventable condition [4,28,29]. It can occur with any kind of nutritional intervention (oral, enteral, or parenteral) [28]. RFS risk predictors have been investigated in many studies, but sensitivity and specificity are low [29,30,31]. Starvation remains the most reliable predictor of RFS [28]. Nutritional risk screening 2002 ≥ 3 points, polymorbidity, older age, and low serum magnesium (<0.7 mmol/L) were found to be risk factors for RFS in many studies [19,20,28,32,33,34,35,36,37,38]. According to the literature and to our long-lasting daily clinical experience, there are many clinical conditions at particular risk of developing RFS (see Table 1). Oncological and geriatric patients are very likely to develop RFS [4,39]. Underlying diseases and conditions affecting nutrient absorption (e.g., short bowel syndrome, bariatric surgery, eating disorders) may be risk factors as well [35]. Moreover, chronic gastrointestinal symptoms (e.g., diarrhea, vomiting) and polypharmacy may increase the risk of RFS [19,20,40]. Additionally, medical therapeutic interventions like hemodialysis or chemotherapy are associated with a high risk of RFS [14,23,26]. Before starting nutritional therapy, it is therefore recommended by the experts’ consensus of Friedli et al. (Figure 2) to assess the patient’s individual risk of RFS and to adapt the nutritional care plan accordingly [19,20,23].

## 5. Diagnostic Procedure

**Figure 3:**
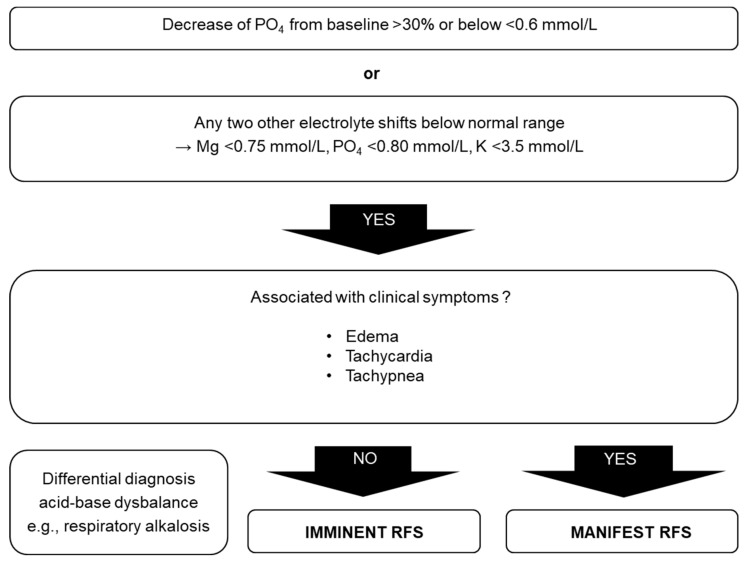
Diagnosis of RFS according to [19], and adapted from Rio et al. [28]. These diagnostic criteria have not been validated in a clinical trial [22]. Used by permission of the Division of Diabetes, Endocrinology, Nutritional Medicine and Metabolism, Prof. Dr. med. Zeno Stanga (2019).

Even though RFS was identified more than 75 years ago, no common definition exists. Therefore, the diagnosis is often delayed or can even be overlooked. Electrolyte imbalance, mainly hypophosphatemia, was used to define RFS in several studies [8,15,41]. Clinical manifestations such as edemas, respiratory failure, or heart failure may occur as a consequence of the electrolyte imbalances, vitamin deficiencies, and fluid overload. The diagnostic procedure proposed by Friedli et al. consists of pathophysiological and clinical characteristics (Figure 3). RFS is probable if the phosphate level in the blood drops >30% under the lower normal value or under 0.6 mmol/L, or if two of the three electrolytes (phosphate, magnesium, and potassium) drop under the normal values within the first 72 h after the start of the replenishment phase in the absence of other possible causes [19,20]. RFS manifests as soon as clinical symptoms occur in addition to electrolyte imbalance [19,20].

## 6. Clinical Management

Each malnourished, catabolic patient should receive the best nutritional support according to the highest quality standards in a timely fashion. A recent randomized controlled trial demonstrated the efficacy of adequate nutritional management [5]. Patients at risk of developing RFS need replenishment of electrolytes and vitamins (especially thiamine) serum levels to help prevent/treat symptoms. In this study, the data from 967 malnourished patients were analyzed for RFS; 141 (14.6%) had confirmed RFS, indicating the high incidence of this metabolic state in medical patients receiving nutritional support [6]. The clinical manifestation can vary from mild forms with limited clinical signs and symptoms to severe forms with potentially lethal complications.

Diverse trials evaluated preventive approaches for RFS, such as substitution of electrolytes, thiamine administration, and hypocaloric feeding. Most studies used for the proposed nutritional management were observational and not interventional, pointing to the overall low level of evidence (see Table 2 for guidelines and Table 3 for trials). From the 45 studies included in the systematic review of Friedli et al. [20], only a few reported on therapeutic strategies to treat RFS; some of them reported phosphate supplementation to be effective. Several studies demonstrated a preventive effect of hypocaloric feeding and a reduced risk of RFS when replacing electrolytes. Moreover, close monitoring of serum electrolytes is a further measure for the reduction of risk of RFS.

**Figure 4:**
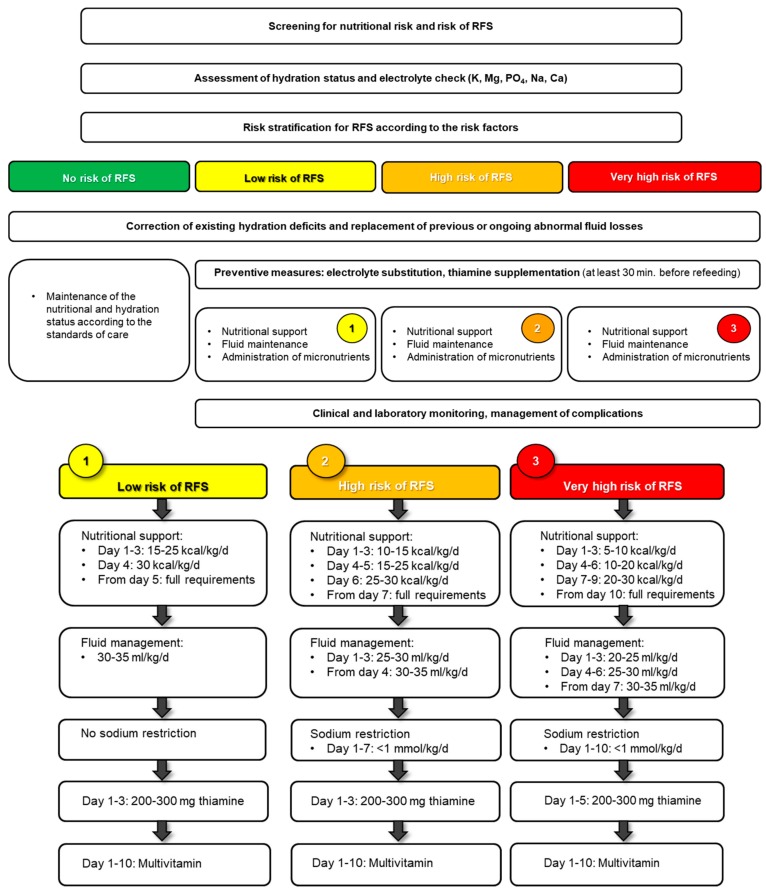
Management of nutritional therapy according to the risk for RFS, after [19]. Used by permission of the Division of Diabetes, Endocrinology, Nutritional Medicine and Metabolism, Prof. Dr. med. Zeno Stanga (2019) [22].

Based on a previously published systematic review, international experts in the field of starvation metabolism and refeeding published a consensus paper [20]. There was a moderate agreement concerning the initial treatment of high-risk patients and prophylactic measures to prevent RFS. For the proposed treatment of imminent or manifest RFS, there was a strong agreement. In this regard, it is advantageous to manage nutritional and fluid intake as proposed in Figure 4.

### 6.1. Macronutrients

Various studies and guidelines have shown a beneficial effect of starting energy intake at a lower rate than generally used, in order to prevent RFS in patients at high risk [12,16,23]. Based on a patient’s individual risk for RFS, energy supply should be initiated at lower levels, starting with an initial amount of 5–15 kcal/kg/day, and increased stepwise depending on the laboratory parameters and clinical situation of the patient [8,19,20,23,52,61,62]. The full energy requirements should be met within 5 to 10 days, depending on the prior risk stratification, using a common nutritional macronutrients composition of 40–60% carbohydrates, 30–40% fats, and 15–20% proteins [12]. In clinically unstable critically ill patients with RFS, lowering the proportion of carbohydrates should be considered.

Nutritional rehabilitation of patients with risk to develop a RFS should be typically started with oral intake of regular food. If the patient cannot eat enough food to meet the energy targets, oral nutritional supplements may be prescribed. Enteral nutrition (tube feeding) is indicated for extremely malnourished patients (e.g., very low BMI) or patients who are unable to consume enough food to reach the energy targets. Parenteral nutrition is indicated when oral and/or enteral nutrition are insufficient or in the case of failure of the gut function. The risk of RFS may be greater with enteral or parenteral feeding compared to oral intake, thus artificial nutrition should be started cautiously at a reduced caloric rate [4,28,29,52,63,64,65,66].

Optimal nutritional support is still controversial and some experts and scientists recommend faster increase in nutritional support to counteract harm associated with malnutrition. Opinions on its management differ, because they are mostly based on personal experience in various populations. At this point, we would like to emphasize that the current review provides important insights into RFS based on a comprehensive literature research and critical appraisal of the evidence. In the light of the current scientific knowledge, it is very likely that there is a need for different intervention approaches adapted to the specific pathologies, e.g., anorexia nervosa.

### 6.2. Fluids

Disturbance of the acid-base balance may cause hypophosphatemia. Acute respiratory alkalosis is for example the most common clinical situation in which hypophosphatemia should be expected in hospitalized patients. The often uncritical use of diuretics (loop and thiazide diuretics) promotes the development of alkalosis through volume reduction and loss of electrolytes (chloride, potassium, magnesium). A decreased volume generates metabolic alkalosis in two ways. The reduction of phosphate is much more pronounced in respiratory alkalosis than in metabolic alkalosis of comparable severity [67,68].

RFS may occur regardless of energy restrictions if fluid balance is disregarded [39]. Hydration deficiencies and abnormal losses (e.g., fever, vomiting, diarrhea) should be addressed at the start of a replenishment phase. The choice of replacement fluid is thereby especially relevant. Balanced solutions should be the preferred option, except when replacing gastric and/or fistula losses over stoma. The fluid prescription should include the daily maintenance requirements plus the water and electrolytes replacement of any losses [69]. In general, fluid intake of 25–35 mL/kg/day is sufficient to maintain an adequate hydration state [69]. The fluid intake through artificial nutrition, infusions, and intravenously administered drugs (mainly antibiotics) should also be taken into account, as well as the salt content (up to 155.2 mmol of Na^+^ in one liter of Ringer’s lactate (Hartmann) solution and 154 mmol of Na^+^ in one liter of isotonic 0.9% NaCl solution). Fluid balance should be corrected cautiously and checked daily. Diuretics, especially specific competitive aldosterone antagonists regulating sodium transport in the kidney, may be useful in case of fluid excess [69].

Particular attention should be paid to the sodium concentration of fluids/products given to patients at (very) high risk for RFS. Sodium restriction (<1 mmol/kg/day) should be considered in the first days after the start of the nutritional therapy in order to avoid fluid overload [12,19,25,47].

### 6.3. Micronutrients

Malnourished patients have depleted intracellular micronutrient stores. After the initiation of nutritional therapy, the intracellular flux of vitamins and electrolytes increases, causing serum levels to drop. It is therefore essential to correct electrolyte levels before initiation of the replenishment phase, with the supplementation of phosphate and thiamine being particularly important [15,19,20]. Prophylactic phosphate supplementation should be undertaken in patients at very high risk for RFS even in the case of normal serum levels to avoid or alleviate the occurrence of RFS, as hypophosphatemia plays a key role in RFS. During starvation, body stores of phosphate decrease, despite normal serum levels. As long as the energy metabolism depends on fat oxidation, phosphate is not required; as soon as the patient resumes carbohydrate intake, the metabolism of glucose uses large amounts of phosphate, thus leading to a drop in serum levels [12,15,64].

The prophylactic supplementation of high-dose thiamine (200–300 mg) at least 30 min before beginning refeeding is fundamental. Vitamins should be supplemented to 200% and the trace elements to 100% of the recommended daily intakes. Electrolytes, especially phosphate, potassium, and magnesium, must be closely monitored and supplemented throughout the refeeding period [12,19,25,47]. Hypokalemia is worsened by concomitant hypomagnesemia, since magnesium is necessary for the sodium-potassium-pump activity and therefore an important factor in the tubular resorption of potassium. Potassium supplementation alone is thus insufficient, and persistently low potassium values despite supplementation can subsequently be rectified only with simultaneous magnesium substitution [70]. Hypocalcemia may cause or further worsen hypophosphatemia [71].

Iron should not be supplemented in the first week after the start of the nutritional therapy, even in the case of manifest iron deficiency. As blood production requires high amounts of potassium, hypokalemia may worsen further. Moreover, parenteral iron supplementation must be considered with caution in malnourished catabolic patients, as it may induce and/or prolong hypophosphatemia [7].

## 7. Monitoring

**Figure 5:**
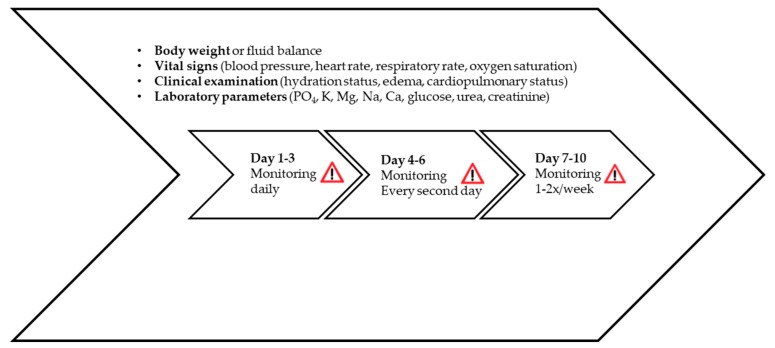
Monitoring of RFS, based on [19]. Used by permission of the Division of Diabetes, Endocrinology, Nutritional Medicine and Metabolism, Prof. Dr. med. Zeno Stanga (2019) [22].

RFS generally occurs within the first 72 h after initiation of nutritional therapy and may progress very rapidly. In the vulnerable phase (up to 10 days), intensive clinical monitoring of vital signs and hydration status, as well as analysis of laboratory parameters, is essential to detect early signs of RFS such as fluid overload and organ failure (mainly kidney) (Figure 5). Body weight and hydration status should be checked on a daily basis, as an increase of 0.3–0.5 kg/day may be an initial sign of pathological fluid retention [12,19,25,47].

Electrocardiogram monitoring is recommended only during the first three days in patients at very high risk of RFS or affected by severe electrolyte imbalances prior to refeeding (K < 2.5 mmol/L, PO_4_ < 0.32 mmol/L, Mg < 0.5 mmol/L), as they may exhibit severe arrhythmia and QT-prolongation, up to Torsades de Pointes [8,12,19,20,23].

Electrolyte substitution respectively supplementation should be initiated or reinforced in case of extracellular electrolyte levels dropping (Table 4). In the case of edema, tachycardia, or tachypnea, symptoms should be treated individually and nutritional therapy should be continued according to the algorithm for the highest risk category [15,19,20].

## 8. Important Clinical Sequelae of Refeeding Syndrome and Management of Complications

RFS may increase rates of morbidity and mortality in severely catabolic patients (Table 5). Malnutrition may result from reduced food intake, reduced absorption of nutrients (e.g., coeliac disease, pancreatitis), or hypermetabolism (e.g., cancer, critical illness, surgery). Mild metabolic imbalances of electrolytes, fluid, and micronutrients are however often asymptomatic but may cause organ dysfunctions and become potentially lethal. Peripheral edema, tachypnea, and tachycardia are the most commonly observed clinical symptoms in patients suffering RFS. It is mandatory to treat these symptoms if they occur, ruling out an eventual lung embolism.

The first step in the management of RFS-related pathological conditions is to anticipate with preventive measures and closely monitor the at-risk patients. The overall objectives in the treatment of RFS complications are to stabilize the patient’s general clinical state, to reverse the medical complications, as well as to restore nutritional needs and weight. The sooner the RFS complications are treated, the lower the risk of damage to patient’s vital organs. The patients with RFS are often dehydrated and require correction of existing hydration deficits and replacement of abnormal fluid losses. Furthermore, electrolytes and vitamins have to be supplemented adequately, as well as any deficiency corrected. The nutritional rehabilitation should be started slowly and adapted to each individual patient. The introduction of carbohydrates in the replenishment phase leads to a quick decrease in renal excretion of sodium and water [21,77]. Patients require a close monitoring of the fluid balance to prevent fluid overload. Such uncontrolled clinical situations may lead quickly to congestive cardiac failure, pulmonary and brain edema, as well as cardiac arrhythmia [78,79]. Too much delivering of glucose in this vulnerable phase leads to hyperglycemia and consequently to osmotic diuresis, dehydration metabolic acidosis, hyperosmotic coma, and ketoacidosis, as well as increased carbon dioxide, hypercapnia, and respiratory failure [77,80,81,82]. Severely malnourished patients may suffer from hematological disorders and moderate to high increase of liver enzymes. The first pathophysiological changes are associated with bone marrow hypoplasia and with gelatinous marrow transformation [83,84]; the second seems to be multicausal and related to an ischemic hepatitis secondary to liver hypoperfusion, to oxidant stress from low glutathione levels, and to starvation-induced autophagy [83,85]. Both pathologies show a marked decrease after a few days during the replenishment phase (hydration and nutritional therapy) and possibly will normalize after the refeeding period [86]. In all these clinical situations, a complications-centric approach to RFS-related complications identifies patients who will benefit most from individual specific interventions and optimizes patient outcomes.

## 9. Outlook

Due to the lack of large randomized trials, the current literature confirms the clinical consequences but not the efficacy of measures used to prevent and treat RFS. A recent secondary analysis of the EFFORT trial showed that RFS has a significant impact on mortality and readmission rate [5,6]. Therefore, prevention, detection, and early treatment of malnourished catabolic medical patients at risk of RFS is essential [25,47,52]. As mentioned before, clinical manifestation can vary from mild to severe, and lethal complications are possible. Therefore, an implementation of the nutritional and fluid intake as proposed in Figure 4 seems opportune. Still, it remains unclear whether RFS is a physiological response or a problem of adaptation to nutritional therapy [19,20]. Thus, further research is needed to determine the optimal rate of energy and fluid increase during refeeding, as well as associated factors.

Many other unresolved issues have not yet been clarified. Does hypoglycemia or hyperglycemia play an important role in the clinical manifestation of RFS? Does insulin therapy influence the risk of RFS? Is RFS caused and/or influenced by the underlying disease [87]? Is there a difference between nutrition-induced hypophosphatemia and RFS? Are there reliable predictors of RFS [31]? For example, increased IGF-1 combined with increased leptin levels is associated with a 30% decrease in the phosphate level within the first 12–36 h after the start of parenteral nutrition [88]. Cytokines may also play an important role in the pathophysiology. Many studies have shown the importance of thiamine supplementation to avoid beriberi disease, whereas the potential action of other vitamins and trace elements in this context is much less investigated [78,89,90].

## 10. Conclusions

Nutritional therapies have shown to be efficacious and efficient, despite the overall low level of evidence. It however hides the risk of RFS in catabolic malnourished patients. RFS is a highly challenging metabolic situation, leading to potentially life-threatening complications with fluid and electrolyte disturbances. RFS should therefore be timely and adequately treated. As nutritional risk is associated with the risk of RFS, awareness of both conditions must be increased among the medical staff in daily clinical practice.

## Figures and Tables

**Table 1:** Clinical conditions at particular risk of developing RFS.

Clinical Conditions	
- Malnourished, catabolic patients - Geriatric patients - Oncologic patients - Trauma patients - Critically ill patients - Hunger strikers or prolonged fasting - Short -bowel syndrome - Bariatric surgery - Anorexia nervosa - Cystic fibrosis	- Chronic wasting disease - Chronic pancreatitis - Chronic infectious disease - Inflammatory bowel syndrome - Liver cirrhosis - Patients with dysphagia - Patients with hemodialysis - Patients with chemotherapy - Patients with chronic alcoholism - Drug dependent patients

**Table 2:** Relevant guidelines and reviews regarding the management of RFS.

Reference	Type of Study	Level of Evidence	Initial Energy/day	Proteins/day	Fluids/day	Vitamins (Before/During)
Solomon et al. 1990 [11]	Review	4	20 kcal/kg	1.2–1.5 g	NR	NR
Dewar et al. 2000 [42]	Review, guidelines	4	20 kcal/kg	NR	NR	Thiamine IV or PO for 2 days
Crook et al. 2001 [8]	Review	4	10 kcal/kg high risk: 5 kcal/kg 50–60% CHO, 15–25% fat	20–30% 1.2–1.5 g	20–30 mL/kg, 0 fluid balance	Thiamine 300 mg IV, than 100 mg daily during refeeding. In addition, Vit B12, Vit B6 and folate
Stroud et al. 2003 [43]	Review	4	10–20 kcal/kg	NR	NR	Thiamine and B vitamins IV for 3 days
Kraft et al. 2005 [44]	Review, guidelines	4	7.5 kcal/kg	NR	<1000 mL/day	Thiamine 50–100 mg IV or 100 mg PO for 5–7 days and multivitamin
NICE 2006 [23]	Review, guidelines	4	10 kcal/kg high risk: 5 kcal/kg	NR	0 fluid balance	Thiamine 200–300 mg PO for 10 days and multivitamin for 10 days
Stanga et al. 2008 [12]	Case series	4	10–15 kcal/kg high risk: 5 kcal/kg 50–60% CHO, 30–40% fat	15–20%	20–30 mL/kg, 0 fluid balance	Thiamine 200–300 mg IV or PO for 3 days and multivitamin for 10 days
Mehanna et al. 2008 [16]	Review	4	10 kcal/kg high risk: 5 kcal/kg	NR	carefully fluid repletion	Thiamine 200–300 mg PO for 10 days and multivitamin for 10 days
Boateng et al. 2010 [15]	Case series	4	10 kcal/kg high risk: 5 kcal/kg 50–60% CHO, 15–25% fat	20–30% 1.2–1.5 g	20–30 mL/kg, 0 fluid balance	Thiamine 300 mg IV, then 100 mg daily during refeeding. In addition, Vit B12, Vit B6 and folate
ESPEN 2019 [45]	Review, guidelines	4	10–15 kcal/kg high risk: 5 kcal/kg 50–60% CHO, 30–40% fat	15–20%	20–30 mL/kg, 0 fluid balance	Thiamine 200–300 mg IV or PO for 3 days and multivitamin for 10 days
Crook et al. 2014 [46]	Review	4	10 kcal/kg high risk: 5 kcal/kg 50–60% CHO, 15–25% fat	20–30% 1.2–1.5 g	20–30 mL/kg, 0 fluid balance	Thiamine 300 mg IV, then 100 mg daily during refeeding. In addition, Vit B12, Vit B6 and folate
Friedli et al. 2017 [20]	Systematic review	3a	10–15 kcal/kg high risk: 5 kcal/kg 50–60% CHO, 30–40% fat	15–20%	20–30 mL/kg, 0 fluid balance	Thiamine 200–300 mg IV or PO for 3 days and multivitamin for 10 days
Friedli et al. 2018 [19]	Systematic review, consensus paper	3a	10–15 kcal/kg high risk: 5 kcal/kg 50–60% CHO, 30–40% fat	15–20%	20–30 mL/kg, 0 fluid balance	Thiamine 200–300 mg IV or PO for 3 days and multivitamin for 10 days

CHO: carbohydrates, IV: intravenous, NR: not reported, PO: per os. Level of evidence after level of evidence for clinical studies from the Oxford centre for evidence-based medicine, http://www.cebm.net; 4 case series (and poor-quality cohort and case-control studies); 3a systematic review (with homogeneity) of case-control studies; 3b individual case-control study.

**Table 3:** Relevant studies regarding the management of RFS.

Reference	Type of Study	Level of Evidence	N	Preventive Medication	Therapeutic Medication	Effectivity
Hofer et al. 2014 [25]	Retrospective study	3b	86	Hypocaloric feeding, restricted fluid administration (0 fluid balance), thiamine 200–300 mg IV or PO for 3 days and multivitamin for 10 days, electrolyte supplementation (unless prefeeding serum levels are high): PO_4_ 0.5–0.8 mmol/kg/day, K 1–2.2 mmol/kg/day, Mg 0.3–0.4 mmol/kg/day	Hypocaloric feeding, restricted fluid administration, electrolytes substitution according to the serum level	Yes
Eichelberger et al. 2014 [47]	Retrospective study	3b	37	Hypocaloric feeding, restricted fluid administration (0 fluid balance), thiamine 200–300 mg IV or PO for 3 days and multivitamin for 10 days, electrolyte supplementation (unless prefeeding serum levels are high): PO_4_ 0.5–0.8 mmol/kg/day, K 1–2.2 mmol/kg/day, Mg 0.3–0.4 mmol/kg/day	Hypocaloric feeding, restricted fluid administration, electrolytes substitution according to the serum level	Yes
Terlevich et al. 2003 [31]	Prospective study	4	30	NR	50 mmol PO_4_ over 24h	Yes
Gonzalez Aviva et al. 1996 [48]	Prospective study	3b	106	PO_4_ supplementation	NR	Yes
Marvin et al. 2008 [49]	Case control study	3b	140	During the first 24 h slow PN regimen providing <70% of protein and calories but >12 mmol PO_4_	NR	Yes
Garber et al. 2011 [50]	Retrospective study	4	40	No effective preventive measures found	NR	No
Coskun et al. 2014 [51]	Retrospective study	4	117	Lower energy intake	NR	No
Doig et al. 2015 [52]	RCT	1b	339	NR	Lower caloric intake	Yes
Whitelaw et al. 2010 [53]	Retrospective study	4	46	Prophylactic administration of PO_4_, lower initial energy intake, monitoring of PO_4_	Supplementation of PO4	Yes
Luque et al. 2007 [54]	Retrospective study	4	11	PO_4_ supplementation, thiamine 3.51 mg/d	NR	Yes
Manning et al. 2014 [55]	Prospective study	2b	36	Repeated electrolyte testing	NR	No
Fan et al. 2004 [33]	Retrospective study	4	158	PO_4_ supplementation	NR	Yes, if PO_4_ <0.30
Gentile et al. 2010 [56]	Retrospective study	4	33	Prophylactic administration of PO_4_ and K, cautious nutritional rehabilitation	NR	Yes
Vignaud et al. 2010 [38]	Retrospective study	4	68	For patients at risk for initial nutritional support 10 kcal/kg/day falling to as low as 5 kcal/kg/day	NR	Yes
Chen et al. 2014 [57]	Retrospective study	4	56	Thiamine and multivitamin supplementation, 15 kcal/kg/day	NR	Yes
Golden et al. 2013 [58]	Retrospective study	4	310	Lower caloric intake	NR	No
Leclerc et al. 2013 [59]	Retrospective study	4	29	Hypocaloric feeding	NR	No
Flesher et al. 2005 [60]	Retrospective study	4	51	Thiamine supplementation, cautious feeding	NR	No
Rio et al. 2013 [28]	Prospective	2b	243	Hypocaloric feeding	NR	No

IV: intravenous, NR: not reported, PO: per os, RCT: randomized controlled trial. Level of evidence after Level of evidence for clinical studies from the Oxford Centre for Evidence-based Medicine, http://www.cebm.net.

**Table 4:** Suggested supplementation regimen [8,12,72,73,74,75,76].

	Potassium	Magnesium	Phosphate
Mild deficiency	3.1–3.5 mmol/L Oral replacement with 20 mmol (as KCl or other salts) OR i.v. replacement with 20 mmol KCl over 4 to 8 h. Check levels the next day.	0.5–0.7 mmol/L Oral replacement with 10–15 mmol MgCl_2_ or Mg-citrate or Mg-L-aspartate Oral Mg should be given in divided doses to minimize diarrhea (absorption process is saturated at about 5–10 mmol Mg)	0.61–0.8 mmol/L Oral replacement with 0.3 mmol/kg/day PO_4_ (divided doses to minimize diarrhea) OR i.v. replacement with 0.3 mmol/kg/day PO_4_ (as K_3_PO_4_ or Na_3_PO_4_) over 8–12 h. Check levels the next day.
Moderate deficiency	2.5–3.0 mmol/L i.v. replacement with 20–40 mmol KCl over 4–8 h. Check levels after 8 h; if not normal, give an additional 20 mmol KCl.		0.32–0.6 mmol/L i.v. replacement with 0.6 mmol/kg/day PO_4_ (as K_3_PO_4_ or Na_3_PO_4_) over 8–12 h. Check levels after 8–12 h and repeat infusion if necessary (max. of 50 mmol PO_4_ in 24 h).
Severe deficiency	<2.5 mmol/L i.v. replacement with 40 mmol KCl over 4–8 h. Check levels after 8 h; if not normal, give an additional 40 mmol KCl.	<0.5 mmol/L i.v. replacement with 20–24 mmol MgSO_4_ (4–6 g) over 4–8 h. Reassess every 8 to 12 h.	<0.32 mmol/L Same replacement therapy as for moderate deficiency.

**Table 5:** Important symptoms and clinical sequelae of RFS (adapted from [15]).

System	Symptoms
Cardiovascular	Tachycardia Arrhythmias Hypotension Congestive heart failure Shock Edemas Sudden death
Gastrointestinal	Maldigestion and malabsorption Vomiting Constipation Abdominal pain
Musculoskeletal	Weakness Myalgia Rhabdomyolysis Osteomalacia
Respiratory	Tachypnea Dyspnea Respiratory failure Ventilator dependency Diaphragm muscle weakness
Neurologic	Anorexia Paresthesia Tremor Wernicke encephalopathy Korsakoff syndrome Ataxia Tetany Delirium Seizures Coma
Metabolic	Hyperglycemia Metabolic alkalosis Metabolic acidosis Respiratory alkalosis Insulin resistance
Hematologic	Thrombocytopenia Hemolysis Anemia Leukocyte dysfunction Decreased 2,3-DPG
Renal	Acute tubular necrosis
Hepatological	Acute liver failure
